# Relationship between disease course in the temporomandibular joints and mandibular growth rotation in patients with juvenile idiopathic arthritis followed from childhood to adulthood

**DOI:** 10.1186/1546-0096-8-13

**Published:** 2010-04-22

**Authors:** MG Fjeld, LZ Arvidsson, H-J Smith, B Flatø, B Øgaard, TA Larheim

**Affiliations:** 1 Department of Orthodontics, Faculty of Dentistry, University of Oslo, Oslo, Norway; 2 Department of Maxillofacial Radiology, Faculty of Dentistry, University of Oslo, Oslo, Norway; 3 Department of Radiology, Rikshospitalet, Oslo University Hospital and University of Oslo, Oslo, Norway; 4 Department of Rheumatology, Rikshospitalet, Oslo University Hospital and University of Oslo, Oslo, Norway

## Abstract

**Objective:**

To investigate the relationship between radiographic JIA disease course in the TMJs and mandibular growth rotation, compared with growth in healthy individuals.

**Methods:**

From a larger series of JIA patients followed from childhood to adulthood, 26 were included; 11 without and 15 with bilateral radiographic TMJ involvement. Joint morphology and function were assessed at baseline, 2-, 4-, 6- and 27 years follow-up. Mandibular growth rotation (anterior, posterior or none) was assessed from cephalometric evaluations at childhood and adulthood, with observations from 16 healthy individuals as controls. TMJ disease course and mandibular growth rotation were assessed independently and their relationship analysed. Non-parametric statistical methods were applied to test differences between groups.

**Results:**

In the normal TMJ group of JIA patients the joint morphology was similar at the follow-ups and all patients had good function both in childhood and in adulthood. The mandibular growth rotation was similar to that of healthy controls, i.e. predominantly in anterior direction. In the abnormal TMJ group different JIA TMJ disease courses were observed and associated with changes in the mandibular growth rotation (p = 0.007).

Progressing JIA TMJ disease course was related to posterior mandibular growth rotation and improving disease course to anterior mandibular growth rotation.

**Conclusion:**

A relationship was found between JIA disease course in the TMJs and mandibular growth rotation, suggesting that a favourable growth could be regained in patients with improvement in TMJ morphology and/or TMJ function. To confirm this, further research on larger patient series is needed.

## Background

Juvenile idiopathic arthritis (JIA) is a heterogeneous group of conditions including seven subtypes according to the ILAR (International League of Associations for Rheumatology) classification [1]. The disease involves persistent inflammation of one or more predominantly large joints, and growth disturbances are characteristically found [2].

The temporomandibular joint (TMJ) is frequently affected in JIA, leading to disturbed mandibular function and growth, with micrognathia being a possible end result [3-5]. Aberrant craniofacial morphology in different age groups has been reported in a number of studies [6-11]. Several TMJ studies of JIA patients have recently been published [12-16], including a review of the literature [17]. However, only a limited number of the studies are longitudinal, focusing on TMJ abnormalities related to craniofacial growth, and most of them show a posterior (dorsal) growth direction of the mandible [18-21]. In one recent longitudinal study anterior growth rotation dominated at a 5-year follow-up examination in patients with improvement of TMJ involvement [22]. Comprehensive studies on TMJ and craniofacial growth abnormalities, following patients from childhood to adulthood, seem to be lacking.

From a group of 103 patients with JIA examined as children [6,23], a follow-up examination of 60 patients as adults was carried out without selection for TMJ and craniofacial findings in childhood [24]. Although normal facial profile was found, the majority of the adult patients with TMJ involvement showed a smaller, more retrognathic mandible and a steeper mandibular plane than healthy controls [5]. The differences in craniofacial morphology between the patients and the controls increased with older age [25]. Further, the frequency of radiographic TMJ involvement increased significantly from childhood to adulthood [24]. Although the development and progression of radiographic TMJ abnormalities was a dominating feature, an evident improvement of radiographic TMJ abnormalities also occurred.

The aim of the present study was to investigate the association between radiographic JIA TMJ disease course and mandibular growth rotation from childhood to adulthood, compared with growth in healthy individuals.

## Methods

### Ethical approvement

The study protocol was approved by the Regional Committee for Medical Research Ethics, Southern Norway, and informed consent was obtained from the participants at the final follow-up examination.

### Subjects

#### JIA patients

The original material comprised 103 consecutive JIA patients (71 girls, 32 boys) initially examined between 1976 and 1979. The mean age at baseline was 9.0 years (range 2.5 - 16.4 years) and the mean age at disease onset was 5.5 years (range 0.8 - 14.5 years). The patients were examined with two-year intervals and followed for either four or six years [6,23].

Sixty patients (mean age 35 years) participated voluntarily in a follow-up examination 23-31 years (mean 27) after baseline [24]. From this group the patients in the present study were selected: 11 (6 females, 5 males) registered without JIA TMJ involvement throughout the entire follow-up period (normal TMJ group), and 15 (11 females, 4 males) registered with bilateral JIA TMJ involvement before 12 years of age (abnormal TMJ group). The presence or absence of JIA TMJ abnormalities was judged by diagnostic imaging (see radiographic TMJ examination section), including computed tomography (CT) and magnetic resonance imaging (MRI) at the final follow-up. Patients with bilateral JIA TMJ involvement after the age of 12, and those with unilateral JIA TMJ involvement or internal TMJ derangement (disk displacement with remodeling and/or osteoarthritis), were excluded. Together with six patients who had undergone orthognatic jaw surgery, a total of 34 of the 60 patients were excluded from the present study.

At the registration in adult age, all patients underwent clinical rheumatological examination and were classified according to the ILAR classification of JIA [1] by one of the authors (BF) with old and new patient files at hand. Disease variables recorded were: number of active joints, number of joints with limited range of motion, physician's overall assessment of disease activity and patient's global assessment of well-being recorded from 100 mm visual analogue scales (VAS) (where 0 indicates no activity or doing very well, and 100 indicates very high activity or doing very poorly), and Health Assessment Questionnaire (HAQ) scores [26].

None of the patients had received local TMJ treatment (steroid injection) in childhood. Three patients in the normal TMJ group and five in the abnormal TMJ group had been subject to orthodontic treatment.

#### Healthy control individuals

Mandibular growth in the JIA patients was compared with growth in healthy subjects from "The University of Oslo Craniofacial Growth Archives" which includes lateral cephalograms and panoramic radiographs of more than 2000 subjects [27]. Sixteen control subjects (11 females, 5 males) followed longitudinally from 9 to 21 years of age with lateral cephalograms, were selected for the present study. Eleven (6 females, 5 males) were matched for age and gender, as children, to the normal TMJ group and 15 (11 females, 4 males) to the abnormal TMJ group. None of the controls had any known joint disease and they had not been subject to orthodontic treatment.

### Radiographic TMJ examination

To evaluate the course of disease in the TMJs two maxillofacial radiologists (LZA, TAL) studied the entire file of each patient, permitting comparison of later sets with earlier sets of radiographs. The examinations at each registration consisted of panoramic and transcranial radiographs at closed and open mouth, and occasional tomograms, as recently described [24]. Attention was paid to the change of mandibular condyle and temporal bone morphology from one registration to another. A classification system for TMJ disease severity based on bone deformity (grade 0-3) was applied [24]: Grade 0: normal joint, Grade 1: small abnormality, Grade 2: moderate abnormality, Grade 3: extensive abnormality. Progressions or improvements within the limit of each grade were also noted. Increasing joint deformation and development or enlargement of bony erosions were interpreted as progressive changes. Normalization of joint morphology and changes to a more clearly defined cortical outline were interpreted as signs of improvement. The CT and MRI examinations at the final follow-up were interpreted by one general and two maxillofacial radiologists (H-JS, LZA, TAL) [28], in order to confirm the presence or absence of TMJ abnormalities.

In addition to joint morphology the joint function, assessed as mandibular condyle translation from closed mouth to maximally opened mouth, was evaluated radiographically. The midpoint of the condyle was identified as previously described [29], and this point was located well beyond, beyond, beneath or behind the point of the lowest contour of the articular eminence [29,30]. Decreased condylar translation was considered as a sign of progression and increased translation as a sign of improvement.

All radiologists had long experience in interpreting TMJ images. They discussed the imaging TMJ diagnosis; morphology and functional capacity, of each case until consensus was met.

### Cephalographic examination

**Figure 1:**
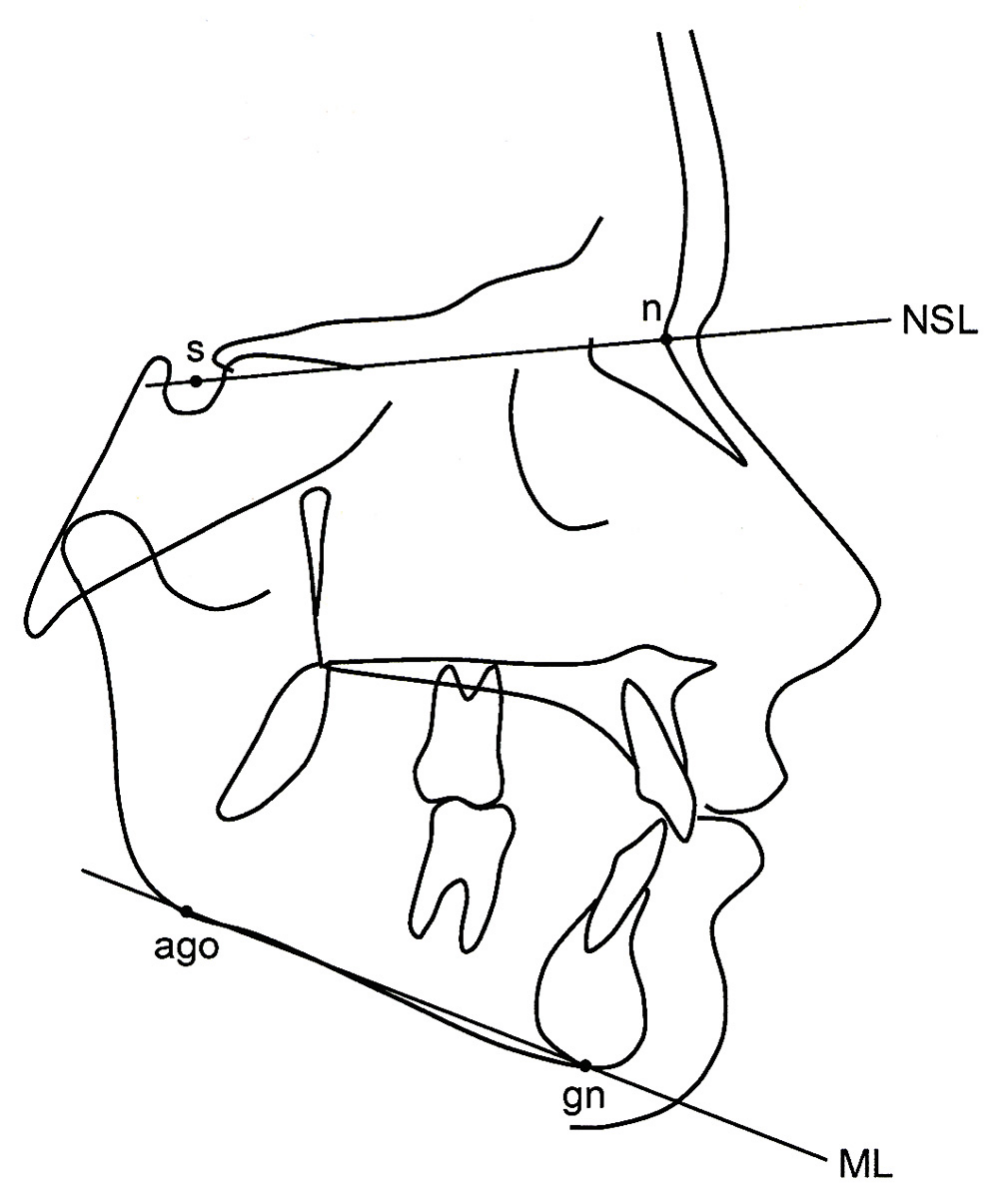
**Cephalometric landmarks and reference lines**. **s **(sella), the midpoint of the cavity of sella turcica; **n **(nasion), the anterior point of the intersection between the nasal and frontal bones; **ago **(anterior gonion), the most inferior point of the gonion;**gn **(gnathion), the most inferior point of the mandibular symphysis; **ML **(mandibular line), tangent to the inferior border of the mandible, from gn to ago; **NSL **(nasion-sella line), the anterior skull basis, from n to s.

The mandibular plane angle (MPA) was measured on standard lateral cephalograms as the angle between the mandibular line (ML) and the nasion-sella line (NSL) (Fig. 1). This angle shows the tilting of the mandible in relation to the anterior cranial base. An overall change in this angle, between the first and final cephalographic evaluation, was chosen as an expression of the direction of the growth and its magnitude; referred to as "mandibular growth rotation" in this article. Anterior (ventral) growth rotation was recorded when the MPA decreased more than two degrees, posterior (dorsal) when the MPA increased more than two degrees, and no growth rotation when the MPA changed less than two degrees.

### TMJ and cephalographic correlation

The TMJ disease course and the mandibular growth were evaluated independently by different observers without knowledge of the result of the others evaluation.

In the analysis of the relationship between JIA TMJ disease course and average mandibular growth, the examinations closest to the patient age of nine years were used and compared with those at the final examinations in adulthood. This age was chosen in order to include patients before the beginning of the pubertal growth spurt, and further, there were 9-year-old healthy controls available for comparison.

### Statistics

Prior to tracing the cephalograms, a measurement error study of the MPA was accomplished by the first author (MF). Twenty randomly selected cephalograms were measured twice with three weeks interval, demonstrating an excellent intrarater reliability analyzed by intraclass correlation, with a value of 0.99. The mean of the two measurements were 27.4° (SD 3.7°) and 27.5° (SD 3.7°), with a p-value of 0.85 (Wilcoxon test). All repeated measurements except one, showed a difference of less than 1° and the majority less than 0.5°. Mann-Whitney test was applied for differences between the normal and abnormal TMJ groups and controls, and for differences in clinical rheumatologic data between the normal and abnormal TMJ groups. Spearman's test was applied to analyze the correlation between TMJ disease course and the mandibular growth rotation. The level of significance was set at p = 0.05.

## Results

### General disease variables

Patients in the abnormal TMJ group were significantly different from those in the normal TMJ group regarding disease onset age and disease duration, number of active joints, joints with limited range of motion, and patients global assessment of well being at final follow-up (Table 1).

**Table 1:** Classification and clinical rheumatological data of 26 JIA patients

	Normal TMJ* group n = 11		Abnormal TMJ group n = 15		p-values
ILAR diagnosis**					
Oligoarthritis	3	(27%)	4	(27%)	
Extended oligoarthritis			4	(27%)	
Polyarthritis, rheumatoid factor positive	2	(18%)	3	(20%)	
Polyarthritis, rheumatoid factor negative			1	(7%)	
Systemic arthritis	3	(27%)	2	(13%)	
Enthesitis related arthritis	2	(18%)	1	(7%)	
Psoriatic arthritis	1	(9%)			
Disease onset age (years)	6.4	± 2.9	3.8	± 1.9	**0.012**
Disease duration at final follow-up (years)	27.4	± 2.3	30.1	± 2.9	**0.017**
Mean age at final follow-up (years)	33.7	± 2.6	33.9	± 3.0	0.72
Disease variables at final follow-up:					
Number of active joints (range 0-69)	1.0	± 1.4	10.4	± 15.0	**0.033**
Number of joints with limited range of motion (range 0-69)	3.2	± 4.7	24.9	± 23.6	**0.009**
Physician's global assessment (0-100 mm VAS)	9.4	± 19.1	24.9	± 30.0	0.064
Patient's global assessment (0-100 mm VAS)	4.8	± 9.6	31.6	± 17.4	**0.001**
Physical function (HAQ disability index, range 0-3)	0.5	± 0.7	0.6	± 0.6	0.23

Values are the number of patients (%), or the mean ± SD

* TMJ = temporomandibular joint

** Patients were classified at the final follow-up with old and new patient files at hand

### TMJ findings

In the 11 JIA patients in the normal TMJ group, the joint morphology changed very little between registrations of the same patients from childhood to adulthood. All patients had a very good TMJ function at baseline and at all follow-ups in childhood; the maximum translatory movement of the mandibular condyle (midpoint) was well beyond the lowest contour of the articular eminence. For six patients this continued into adulthood, and for three the condyles were positioned beneath the eminence at maximally opened mouth. For the remaining two patients functional images at the final examination were missing.

In the 15 patients in the abnormal TMJ group, five courses of disease in the TMJs were found: A) consistently progressing - when the grade increased from 1 to 3 (n = 1); B) varying - when the disease both progressed and improved during the observation time, either by changing grades, or when one joint progressed and the contralateral improved (n = 2); C) stable - when there were no differences in grade and no improvement or progression within grades (n = 6); D) slightly improving - when the TMJ grade improved from grade 3 to 2, or improving within grade 2 including improvement in function (n = 4); E) greatly improving - when the TMJ grade improved from 3 or 2 to 1 (n = 2). The maximum translatory movement of the mandibular condyle varied between patients and within patients between follow-ups.

### Mandibular growth rotation and mandibular plane angle (MPA)

**Figure 2:**
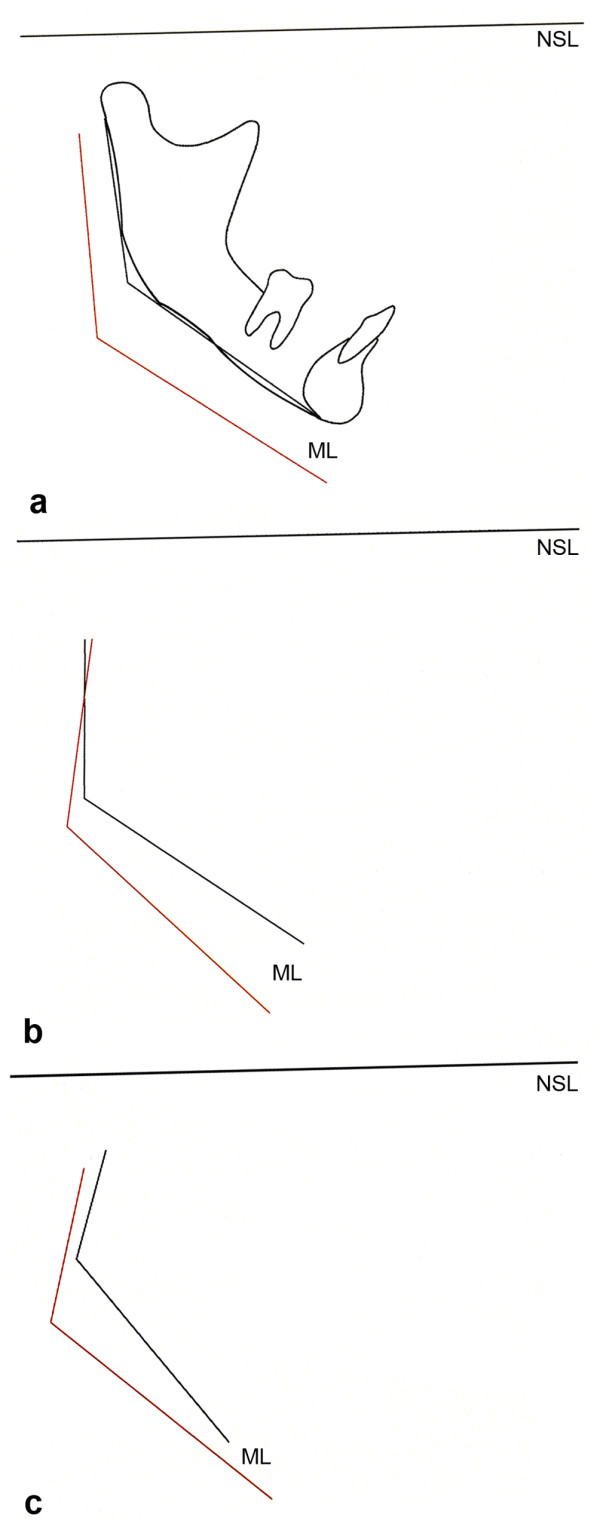
**Mandibular growth rotation in three JIA patients**. Schematic drawings of the change in the mandibular plane angle; angle between the mandibular plane (ML) and the cranial base (NSL), from childhood to adulthood, compare Fig. 1. (a) Anterior growth rotation (3 degrees) from 11 yrs (black line) to 33 yrs (red line) in the same patient as shown in Fig. 3. (b) Posterior growth direction (9 degrees) from 10 yrs (black line) to 38 yrs (red line) in the same patient as shown in Fig. 4. (c) Anterior growth direction (12 degrees) from 9 yrs (black line) to 33 yrs (red line) in the same patient as shown in Figs. 5, 6.

In the total series of 26 JIA patients, 14 showed an anterior mandibular growth rotation, nine showed no growth rotation, and three patients showed a posterior growth rotation (Fig. 2). Large variations in magnitude of growth were observed between the patients.

The mean MPA was larger in the abnormal TMJ group than in the normal TMJ group both as children (p = 0.002) and as adults (p = 0.001) (Table 2). Comparisons between the abnormal TMJ group and the healthy controls revealed similar differences in the MPA as children (p = 0.001) and as adults (p = 0.001). No significant differences in MPA were found between the normal TMJ group and the healthy controls neither as children (p = 0.62) nor as adults (p = 0.77). All groups showed a mean anterior mandibular growth direction. The smallest magnitude of growth (less than two degrees) was found in the abnormal TMJ group, but no statistical differences were found (Table 2).

**Table 2:** Cephalometric values in 26 JIA patients and corresponding healthy controls

		Normal TMJ* group (n = 11)		Controls (n = 11)		Abnormal TMJ group (n = 15)		Controls (n = 15)		p-values		
										Abnormal TMJ vs normal TMJ	Normal TMJ vs control	Abnormal TMJ vs control
**Variable**		**Mean**	**SD**	**Mean**	**SD**	**Mean**	**SD**	**Mean**	**SD**			

Age at examination in childhood	years	9.0	1.1	8.8	0.4	9.6	1.2	8.9	0.5	0.26	0.67	0.16
Disease duration at examination in childhood	years	2.6	2.3			5.7	2.0			**0.003**		
MPA**, children	degrees	31.3	4.0	31.1	4.1	38.2	5.7	30.6	4.8	**0.002**	0.62	**0.001**
MPA, adults	degrees	27.7	5.6	27.1	6.6	36.5	5.2	26.7	7.0	**0.001**	0.77	**0.001**
Diff*** in MPA	degrees	-3.6	3.3	-4.0	3.6	-1.7	5.5	-3.9	3.4	0.14	0.72	0.10

* TMJ = Temporomandibular joint

** MPA = Mandibular plane angle

*** Difference in MPA = MPA adults - MPA children. Negative value = Anterior growth direction of the mandible

### Association between TMJ findings and mandibular growth rotation

**Figure 3:**
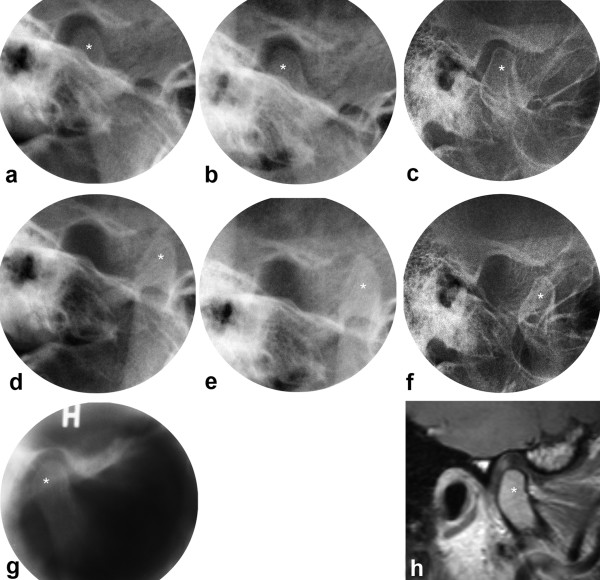
**JIA patient with normal TMJs**. Transcranial radiographs at closed (a,b,c) and maximally opened mouth (d,e,f), including tomogram (g) and MRI (h) at closed mouth. One joint shown from 11 to 33 yrs with normal anatomical structures and normal mandibular condyle translation at baseline (a,d,g), 2 yrs follow-up (b,e) and final follow-up (c,f,h). * = mandibular condyle. Mandibular growth rotation shown in Fig. 2a.

In the healthy controls and in the normal TMJ group, the majority showed a decreasing MPA (Table 3). A typical case from the normal TMJ group is illustrated in Figs. 2a, 3.

**Table 3:** JIA disease course in the TMJs and mandibular growth rotation; rotation in controls for comparison

	MPA* decrease more than 2 degrees	MPA change less than 2 degrees	MPA increase more than 2 degrees
Healthy control group (n = 16)	10	6	
Normal TMJ** group (n = 11)	8	3	
Abnormal TMJ group (n = 15)			
- Consistently progressing (n = 1)			1
- Varying (n = 2)		1	1
- Stable (n = 6)	2	3	1
- Slightly improving (n = 4)	2	2	
- Greatly improving (n = 2)	2		

* MPA = Mandibular plane angle

** TMJ = Temporomandibular joint

**Figure 4:**
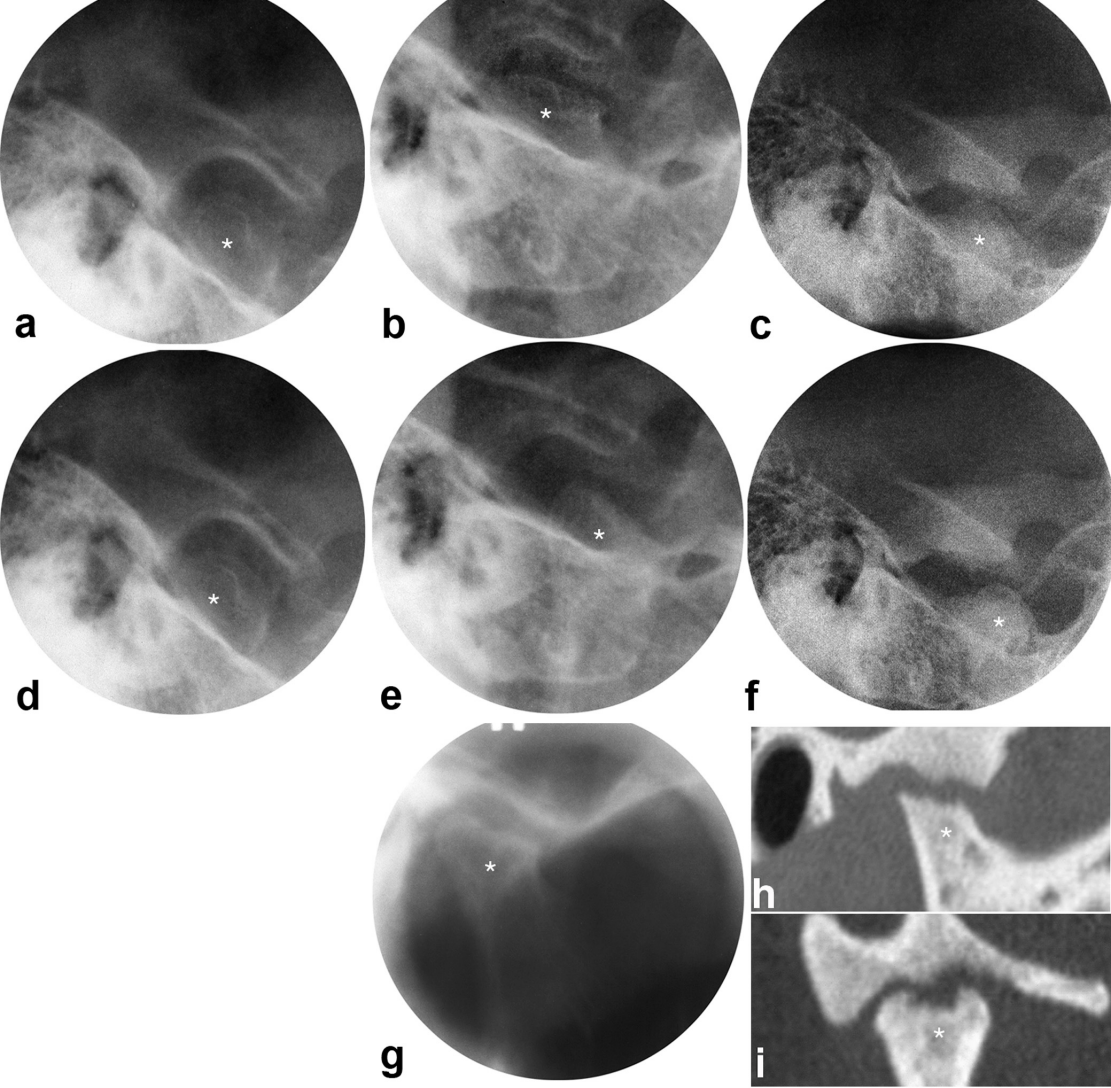
**JIA patient with consistently progressing disease course in the TMJs**. Transcranial radiographs at closed (a,b,c) and maximally opened mouth (d,e,f), including tomogram (g), and oblique sagittal (h) and oblique coronal (i) CT at closed mouth. One joint shown from 10 to 38 yrs with small bone abnormality and severely impaired condyle translation at baseline (a, d), moderate abnormality at 2 yrs follow-up (b,e,g) and extensive abnormality at final follow-up (c,f,h,i). * = mandibular condyle. Mandibular growth rotation shown in Fig. 2b.

**Figure 5:**
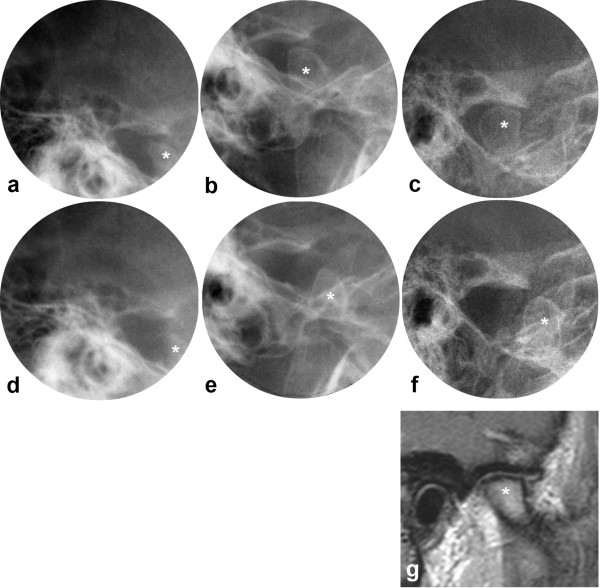
**JIA patient with greatly improving disease course in the TMJs, right joint**. Transcranial radiographs at closed (a,b,c) and maximally opened mouth (d,e,f), including MRI (g) at closed mouth. Right joint shown from 5 to 33 yrs with extensive bone abnormality and severely impaired condyle translation at baseline (a,d), improved condyle morphology and translation at 2 yrs follow-up (b,e), and further improvement of morphology and translation at final follow-up (c,f,g). Note the normalization of the condyle position in the fossa at closed mouth between the first and last examinations. * = mandibular condyle. Mandibular growth rotation shown in Fig. 2c.

**Figure 6:**
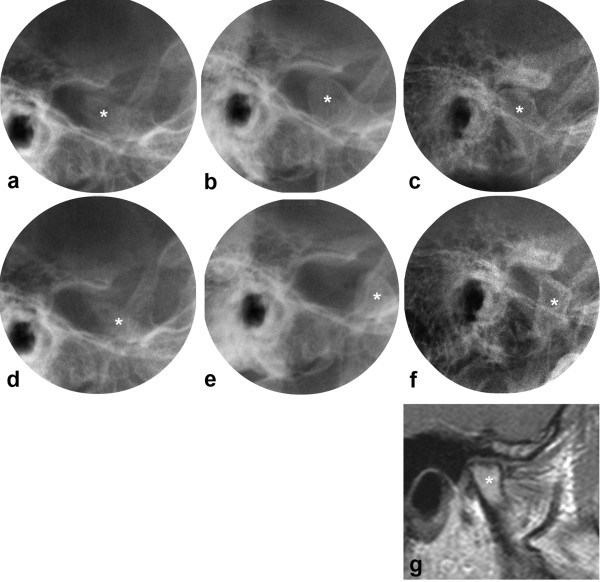
**JIA patient with greatly improving disease course in the TMJs, left joint (same patient as in Fig. 5)**. Transcranial radiographs at closed (a,b,c) and maximally opened mouth (d,e,f), including MRI (g) at closed mouth. Left joint shown from 5 to 33 yrs with extensive bone abnormality, anterior position of the condyle in the fossa at closed mouth and severely impaired condyle translation at baseline (a,d), improved condyle morphology and translation at 2 yrs follow-up (b,e), and further improvement of joint morphology and condyle position at final follow-up (c,f,g). * = mandibular condyle. Mandibular growth rotation shown in Fig. 2c.

In the abnormal TMJ group, three patients had an increasing MPA, while the remaining 12 were distributed equally between a decreasing MPA and no change in the MPA (Table 3). Differences in growth rotation were observed with the different types of disease courses in the TMJs and statistical analysis showed a correlation coefficient of 0.66 (p = 0.007). The largest difference was demonstrated between the patient with consistently progressing disease course and one patient with greatly improving disease course. The first showed a posterior growth rotation of nine degrees (Figs. 2b, 4), and the latter an anterior growth rotation of 12 degrees (Figs. 2c, 5, 6), both with a MPA larger than found in healthy individuals throughout the observations.

In the patients with varying, stable or slightly improving disease course the mandibular growth rotation was less evident, except for one patient with an anterior growth of 12 degrees.

## Discussion

In the present study the JIA course in the TMJs was related to the growth rotation of the mandible. It is the first study that analyzes JIA patients with and without TMJ involvement from childhood to adulthood, using growth in healthy individuals as control.

In the patient group with normal TMJs as judged from conventional radiographs throughout the observation period and verified by CT and MRI at the final follow-up, the joint morphology was similar at all examinations as recently demonstrated in healthy controls [24]. The mandibular growth pattern was similar to that of the controls in accordance with the predominantly anterior growth direction described in healthy individuals [31]. In the present series also the MPA itself was similar in the normal TMJ group and the healthy controls, both as children and as adults.

In the abnormal TMJ group 20% of the patients showed a posterior mandibular growth rotation and 40% showed an anterior growth rotation. As a consequence the mean mandibular growth in the abnormal TMJ group had a slightly anterior direction. For comparison, 73% in the normal TMJ group and 63% in the healthy controls showed an anterior mandibular growth rotation (Table 3). Most previous longitudinal studies on craniofacial growth in JIA patients with TMJ involvement have shown a posterior growth direction [18-21], except for the study by Twilt et al. [22], reporting an anterior growth rotation in patients with improving TMJ findings. In the present study, the lack of difference in mean growth magnitude between abnormal and normal TMJ groups and controls could be explained by the great variations within the groups. Also, many of our patients had JIA TMJ involvement and a large MPA already at inclusion in the present study. If much of the negative effect on the mandibular growth rotation had occurred early, this would not have been registered by our study design. Since many patients show stable or improving conditions we may have missed the initial progression of the disease. Ideally, the patients should have been followed from an earlier age in order to record the full impact of TMJ involvement on mandibular growth rotation.

A posterior mandibular growth direction was found among those with a consistently progressing, varying, or stable disease course in the TMJs, but was not found among those with an improving disease course. On the contrary, four of those had an anterior growth direction. The only others with such favourable mandibular growth direction were found among those with a stable disease course.

Those with a posterior growth direction had severe bilateral TMJ involvement, grade 2 or 3. This is in accordance with two case reports on JIA children [19,20] as well as a study on micrognathic adults [29].

All three patients with a posterior growth direction in the present study had impaired jaw function bilaterally. As children, their condyles were located either behind or beneath the articular eminence at maximally opened mouth. The reduced translation is in accordance with observations in children with JIA [32] and in adults with micrognathia [29], both studies using healthy individuals as controls. It should be noted that the mandibular condyle translation in healthy children usually is pronounced [32], with the condyle moving well beyond the articular eminence. Such observations were made in the patients without TMJ involvement in the present study (Fig. 3). With increasing age the condyle translation in healthy individuals will be less pronounced when related to the articular eminence (Fig. 3), and a location beneath the eminence at maximally opened mouth is considered within the normal range [29].

Six patients with evident JIA TMJ abnormalities had an anterior mandibular growth direction. This favourable growth pattern could be explained by an improving TMJ disease course in four of these patients, in accordance with Twilt et al. [22]. In our study the function appeared to follow the severity of disease in the TMJ at the different registrations; an improved joint morphology was accompanied by an improved condyle translation. One patient with a pronounced anterior mandibular rotation of 12 degrees had a stable TMJ condition with good function throughout the observation period. We believe the sustained good joint function played a role in the favourable mandibular growth rotation of this patient.

Another explanation for a favourable mandibular growth rotation may be the development of hyperplastic mandibular condyles and/or articular eminence/fossa with an overgrowth in craniocaudal direction. One patient with a normal craniofacial profile in adulthood but no registered mandibular rotation had developed extensive bone apposition of the articular eminence/fossa after severe JIA involvement early in childhood [5,28]. It is reasonable to believe that the pronounced overgrowth/remodeling had prevented a posterior mandibular growth direction.

Despite the low number of patients, a correlation was found; the more progressive the JIA TMJ disease course, the greater the risk for a posterior mandibular growth direction and, the more improving the disease course, the greater the chance for an anterior mandibular growth direction.

The present study demonstrated the potential and dynamics of mandibular growth. Even in patients with early severe JIA TMJ involvement, normal growth rotation of the mandible occurred when the TMJ arthritis improved (Figs 2c, 5, 6). Early treatment must surely be of utmost importance in JIA patients, suppressing the disease and maintaining normal growth.

The low disease onset age in the abnormal TMJ group substantiates the observations made by Argyropoulou et al. [15]. This group also had a generally more aggressive disease course than the normal TMJ group. The medical management of children in the 1970s and 1980s, when these patients were diagnosed and treated, was different from today's regimen [33,34]. At that time the general treatment was initiated with NSAIDs and later supplemented with DMARDs and/or corticosteroids. Today the medical treatment is much more aggressive with earlier use of methotrexate and new biologic agents. Further, the treatment is adjusted to JIA subtype. Hopefully, today's treatment will have a positive impact on the craniofacial development of patients with early TMJ involvement as suggested by Ince et al. [35], but further studies are needed to confirm such an effect. Steroid injections have been reported to be a valuable treatment in children with TMJ involvement [36-38], but also here, further studies are needed to demonstrate its effect on craniofacial growth. A study on rabbits have shown a negative effect on mandibular growth after steroid injection in the TMJ [39].

Five patients with bilateral TMJ involvement and a history of orthognathic jaw surgery were excluded from the study. They represented the more extreme cases in mandibular growth and if they had been included, a greater difference between patients with normal and abnormal TMJs would probably have been the result. Pre-operative cephalograms were not available and made such analysis impossible.

Bilateral TMJ involvement before 12 years of age was chosen in order to include the growth spurt, and thereby observe the patients during the period of most extensive growth. Pearson and Rönning [40] found that when TMJ involvement occurred after the age of 12, craniofacial growth disturbances were rare. Patients with unilateral TMJ involvement were excluded from the present study as the healthy side would camouflage the affected side when measuring the mandibular plane angle. The prospect of this study was to explore possible effects of JIA TMJ disease course on mandibular growth, rather than to report the frequency of such findings.

The mandibular plane angle was used as an expression of the mandibular growth rotation in relation to the anterior cranial base. According to Skieller et al. [41] this angle can explain about 50% of the variability in the magnitude of mandibular growth. Development of antegonial notching of the lower mandibular border, leading to a reduced MPA, may be seen in JIA patients [7,21]. This would increase the risk of underestimating the posterior mandibular growth rotation. However, in the present study only one of the six patients with an anterior rotation and one of the six patients without growth rotation had evident antegonial notching bilaterally. Thus, the MPA seemed to be a reliable estimate for the growth rotation of the mandible.

The mean age of the healthy adult controls was more than 10 years younger than that of the JIA patients. This difference was probably without consequence since only small changes in the MPA have been observed in adults between 22 and 33 years of age [42].

Longitudinal studies of greater series of JIA patients are needed to validate the different disease courses in the TMJ and to assess the association with the craniofacial development.

## Conclusions

Despite the low number of patients, a relationship was found between JIA disease course in the TMJs and mandibular growth rotation, suggesting that a favourable growth could be regained in patients with improvement in TMJ morphology and/or function.

## Abbreviations

JIA: Juvenile Idiopathic Arthritis; ILAR: International League of Associations for Rheumatology; TMJ: Temporomandibular Joint; CT: Computed Tomography; MRI: Magnetic Resonance Imaging; MPA: Mandibular Plane Angle.

## Competing interests

The authors declare that they have no competing interests.

## Authors' contributions

MF, LZA, BØ and TAL designed the study. MF performed the evaluations of the lateral cephalograms. LZA, H-JS and TAL performed the evaluations of the TMJ images. BF performed the clinical rheumatological examinations with subgrouping of the patients. MF drafted the initial manuscript. All authors have made significant contributions to the manuscript regarding content. All authors have read and approved the final manuscript.

## References

[B1] Petty RE Southwood TR Manners P Baum J Glass DN Goldenberg J He X Maldonado-Cocco J Orozco-Alcala J Prieur AM Suarez-Almazor ME Woo P International League of Associations for Rheumatology classification of juvenile idiopathic arthritis: second revision, Edmonton, 2001 J Rheumatol 2004 31 390 392 14760812

[B2] Cassidy JT Petty RE Cassidy JT, Petty RE, Laxer RM, Lindsley CB Chronic arthritis in childhood Textbook of pediatric rheumatology 2005 5 Philadelphia: Elsevier Saunders 206 260

[B3] Odenrick L Potential micrognathia in children with juvenile rheumatoid arthritis Trans Eur Orthod Soc 1977 207 216

[B4] Larheim TA Haanaes HR Micrognathia, temporomandibular joint changes and dental occlusion in juvenile rheumatoid arthritis of adolescents and adults Scand J Dent Res 1981 89 329 338 6947392 10.1111/j.1600-0722.1981.tb01690.x

[B5] Arvidsson LZ Fjeld M Smith H-J Flatø B Øgaard B Larheim TA Craniofacial growth disturbance is related to temporomandibular joint abnormality in patients with juvenile idiopathic arthritis, but normal facial profile was also found at the 27-year follow-up Scand J Rheumatol in press 10.3109/03009741003685624 20615158

[B6] Stabrun AE Larheim TA Höyeraal HM Rösler M Reduced mandibular dimensions and asymmetry in juvenile rheumatoid arthritis. Pathogenetic factors Arthritis Rheum 1988 31 602 611 10.1002/art.1780310504 3377867

[B7] Rönning O Barnes SA Pearson MH Pledger DM Juvenile chronic arthritis: a cephalometric analysis of the facial skeleton Eur J Orthod 1994 16 53 62 8181551 10.1093/ejo/16.1.53

[B8] Kjellberg H Fasth A Kiliaridis S Wenneberg B Thilander B Craniofacial structure in children with juvenile chronic arthritis (JCA) compared with healthy children with ideal or postnormal occlusion Am J Orthod Dentofacial Orthop 1995 107 67 78 10.1016/S0889-5406(95)70158-3 7817963

[B9] Sidiropoulou-Chatzigianni S Papadopoulos MA Kolokithas G Dentoskeletal morphology in children with juvenile idiopathic arthritis compared with healthy children J Orthod 2001 28 53 58 10.1093/ortho/28.1.53 11254804

[B10] Twilt M Schulten AJ Nicolaas P Dülger A van Suijlekom-Smit LW Facioskeletal changes in children with juvenile idiopathic arthritis Ann Rheum Dis 2006 65 823 825 10.1136/ard.2005.042671 16699052 PMC1798159

[B11] Billiau AD Hu Y Verdonck A Carels C Wouters C Temporomandibular joint arthritis in juvenile idiopathic arthritis: prevalence, clinical and radiological signs, and relation to dentofacial morphology J Rheumatol 2007 34 1925 1933 17696265

[B12] Weiss PF Arabshahi B Johnson A Bilaniuk LT Zarnow D Cahill AM Feudtner C Cron RQ High prevalence of temporomandibular joint arthritis at disease onset in children with juvenile idiopathic arthritis, as detected by magnetic resonance imaging but not by ultrasound Arthritis Rheum 2008 58 1189 1196 10.1002/art.23401 18383394

[B13] Pedersen TK Kuseler A Gelineck J Herlin T A prospective study of magnetic resonance and radiographic imaging in relation to symptoms and clinical findings of the temporomandibular joint in children with juvenile idiopathic arthritis J Rheumatol 2008 35 1668 1675 18634141

[B14] Twilt M Schulten AJ Verschure F Wisse L Prahl-Andersen B van Suijlekom-Smit LW Long-term followup of temporomandibular joint involvement in juvenile idiopathic arthritis Arthritis Rheum 2008 59 546 552 10.1002/art.23532 18383409

[B15] Argyropoulou MI Margariti PN Karali A Astrakas L Alfandaki S Kosta P Siamopoulou A Temporomandibular joint involvement in juvenile idiopathic arthritis: clinical predictors of magnetic resonance imaging signs Eur Radiol 2009 19 693 700 10.1007/s00330-008-1196-2 18958475

[B16] Müller L Kellenberger CJ Cannizzaro E Ettlin D Schraner T Bolt IB Peltomäki T Saurenmann RK Early diagnosis of temporomandibular joint involvement in juvenile idiopathic arthritis: a pilot study comparing clinical examination and ultrasound to magnetic resonance imaging Rheumatology (Oxford) 2009 48 680 5 10.1093/rheumatology/kep068 19386819 PMC2681286

[B17] Ringold S Cron RQ The temporomandibular joint in juvenile idiopathic arthritis: frequently used and frequently arthritic. (Review) Pediatr Rheumatol Online J 2009 7 11 10.1186/1546-0096-7-11 19480670 PMC2694194

[B18] Larheim TA Haanaes HR Ruud AF Mandibular growth, temporomandibular joint changes and dental occlusion in juvenile rheumatoid arthritis. A 17-year follow-up study Scand J Rheumatol 1981 10 225 233 10.3109/03009748109095303 7291954

[B19] Bjork A Skieller V Normal and abnormal growth of the mandible. A synthesis of longitudinal cephalometric implant studies over a period of 25 years Eur J Orthod 1983 5 1 46 6572593 10.1093/ejo/5.1.1

[B20] Kreiborg S Bakke M Kirkeby S Michler L Vedtofte P Seidler B Møller E Facial growth and oral function in a case of juvenile rheumatoid arthritis during an 8-year period Eur J Orthod 1990 12 119 134 2351196 10.1093/ejo/12.2.119

[B21] Stabrun AE Impaired mandibular growth and micrognathic development in children with juvenile rheumatoid arthritis. A longitudinal study of lateral cephalographs Eur J Orthod 1991 13 423 434 1817067 10.1093/ejo/13.6.423

[B22] Twilt M Schulten AJM Prahl-Andersen B van Suijlekom-Smit LWA Long-term Follow-up of Craniofacial Alterations in Juvenile Idiopathic Arthritis Angle Orthod 2009 79 1057 1062 10.2319/093008-511R.1 19852594

[B23] Larheim TA Hoyeraal HM Stabrun AE Haanaes HR The temporomandibular joint in juvenile rheumatoid arthritis. Radiographic changes related to clinical and laboratory parameters in 100 children Scand J Rheumatol 1982 11 5 12 10.3109/03009748209098105 7063812

[B24] Arvidsson LZ Flatø B Larheim TA Radiographic TMJ abnormalities in patients with juvenile idiopathic arthritis followed for 27 years Oral Surg Oral Med Oral Pathol Oral Radiol Endod 2009 108 114 123 10.1016/j.tripleo.2009.03.012 19540449

[B25] Fjeld MG Arvidsson LZ Stabrun AE Birkeland K Larheim TA Øgaard B Average craniofacial development from 6 to 35 years of age in a mixed group of patients with juvenile idiopathic arthritis Acta Odontol Scand 2009 67 153 160 10.1080/00016350902740506 19241184

[B26] Fries JF Spitz P Kraines RG Holman HR Measurement of patient outcome in arthritis Arthritis Rheum 1980 23 137 145 10.1002/art.1780230202 7362664

[B27] el-Batouti A Ogaard B Bishara SE Longitudinal cephalometric standards for Norwegians between the ages of 6 and 18 years Eur J Orthod 1994 16 501 509 7720795 10.1093/ejo/16.6.501

[B28] Arvidsson LZ Smith H-J Flatø B Larheim TA Temporomandibular joint findings in adults with long-standing juvenile idiopathic arthritis: CT and MR imaging assessment Radiology in press 10.1148/radiol.10091810 20574096

[B29] Larheim TA Haanaes HR Dale K Radiographic temporomandibular joint abnormality in adults with micrognathia and juvenile rheumatoid arthritis Acta Radiol Diagn 1981 22 495 504 10.1177/028418518102200413 7331861

[B30] Ahmad M Hollender L Anderson Q Kartha K Ohrbach R Truelove EL John MT Schiffman EL Research diagnostic criteria for temporomandibular disorders (RDC/TMD): development of image analysis criteria and examiner reliability for image analysis Oral Surg Oral Med Oral Pathol Oral Radiol Endod 2009 107 844 860 10.1016/j.tripleo.2009.02.023 19464658 PMC3139469

[B31] Karlsen AT Craniofacial growth differences between low and high MP-SN angle males: a longitudinal study Angle Orthod 1995 65 341 350 8526293 10.1043/0003-3219(1995)065<0341:CGDBLA>2.0.CO;2

[B32] Stabrun AE Larheim TA Rosler M Haanaes HR Impaired mandibular function and its possible effect on mandibular growth in juvenile rheumatoid arthritis Eur J Orthod 1987 9 43 50 3470185 10.1093/ejo/9.1.43

[B33] Hashkes PJ Laxer RM Medical treatment of juvenile idiopathic arthritis. (Review) JAMA 2005 294 1671 84 10.1001/jama.294.13.1671 16204667

[B34] Hayward K Wallace CA Recent developments in anti-rheumatic drugs in pediatrics: treatment of juvenile idiopathic arthritis Arthritis Res Ther 2009 11 216 10.1186/ar2619 19291269 PMC2688259

[B35] Ince DO Ince A Moore TL Effect of methotrexate on the temporomandibular joint and facial morphology in juvenile rheumatoid arthritis patients Am J Orthod Dentofacial Orthop 2000 118 75 83 10.1067/mod.2000.104953 10893476

[B36] Cahill AM Baskin KM Kaye RD Arabshahi B Cron RQ Dewitt EM Bilaniuk L Towbin RB CT-guided percutaneous steroid injection for management of inflammatory arthropathy of the temporomandibular joint in children AJR Am J Roentgenol 2007 188 182 186 10.2214/AJR.04.1103 17179362

[B37] Arabshahi B Dewitt EM Cahill AM Kaye RD Baskin KM Towbin RB Cron RQ Utility of corticosteroid injection for temporomandibular arthritis in children with juvenile idiopathic arthritis Arthritis Rheum 2005 52 3563 3569 10.1002/art.21384 16255045

[B38] Ringold S Torgerson TR Egbert MA Wallace CA Intraarticular corticosteroid injections of the temporomandibular joint in juvenile idiopathic arthritis J Rheumatol 2008 35 1157 1164 18398938

[B39] Stoustrup P Kristensen KD Küseler A Gelineck J Cattaneo PM Pedersen TK Herlin T Reduced mandibular growth in experimental arthritis in the temporomandibular joint treated with intra-articular corticosteroid Eur J Orthod 2008 30 111 119 10.1093/ejo/cjm096 18209214

[B40] Pearson MH Rönning O Lesions of the mandibular condyle in juvenile chronic arthritis Br J Orthod 1996 23 49 56 8652498 10.1179/bjo.23.1.49

[B41] Skieller V Björk A Linde-Hansen T Prediction of mandibular growth rotation evaluated from a longitudinal implant sample Am J Orthod 1984 86 359 370 10.1016/S0002-9416(84)90028-9 6594058

[B42] Bondevik O Growth changes in the cranial base and the face: a longitudinal cephalometric study of linear and angular changes in adult Norwegians Eur J Orthod 1995 17 525 532 8682170 10.1093/ejo/17.6.525

